# Discriminative and predictive properties of disease-specific and generic health status indexes in elderly COPD patients

**DOI:** 10.1186/1471-2466-8-14

**Published:** 2008-08-13

**Authors:** Maria E Conte, Claudio Pedone, Francesco Forastiere, Vincenzo Bellia, Raffaele Antonelli-Incalzi

**Affiliations:** 1 Area di Geriatria, Università Campus Biomedico, Roma, Italy; 2 Dipartimento di Epidemiologia, ASL Roma E, Roma, Italy; 3 Dipartimento di Medicina, Pneumologia, Fisiologia e Nutrizione Umana (DIMPEFINU), Università di Palermo, Palermo, Italy; 4 Fondazione S. Raffaele – Cittadella Della Carità, Taranto, Italy

## Abstract

**Background:**

The association between bronchial obstruction severity and mortality in Chronic Obstructive Pulmonary Disease (COPD) is well established, but it is unknown whether disease-specific health status measures and multidimensional assessment (MDA) have comparable prognostic value.

**Methods:**

We analyzed data coming from the Salute Respiratoria nell'Anziano (Respiratory Health in the Elderly – SaRA) study, enrolling elderly people attending outpatient clinics for respiratory and non-respiratory problems. From this population we selected 449 patients with bronchial obstruction (77.3% men, mean age 73.1). We classified patients' health status using tertiles of the Saint George Respiratory Questionnaire (SGRQ) and a MDA including functional (the 6' walking test, WT), cognitive (Mini-Mental State Examination, MMSE) and affective status (Geriatric Depression Scale, GDS). The agreement of the classification methods was calculated using the kappa statistic, and survival associated with group membership was evaluated using survival analysis.

**Results:**

Pulmonary function, expressed by the FEV1, worsened with increasing SGRQ or MDA scores. Cognitive function was not associated with the SGRQ, while physical performance and mood status were impaired only in the highest tertile of SGRQ. A poor agreement was found between the two classification systems tested (k = 0.194). Compared to people in the first tertile of SGRQ score, those in the second tertile had a sex-adjusted HR of 1.22 (0.75 – 1.98) and those in the third tertile of 2.90 (1.92 – 4.40). The corresponding figures of the MDA were 1.49 (95% CI 1.02 – 2.18) and 2.01 (95% CI: 1.31 – 3.08). After adjustment for severity of obstruction, only a SGRQ in the upper tertile was associated with mortality (HR: 1.86; 95% CI: 1.14 – 3.02).

**Conclusion:**

In elderly outpatients with mild-moderate COPD, a disease-specific health status index seems to be a better predictor of death compared to a MDA.

## Background

The rapid increase of COPD prevalence has important implications for health care and costs [1,2], although most of these effects are attributable to comorbid diseases [3,4]. Given that the mean age of COPD patients is continuously rising [5] and older age is associated with comorbidity [6], we expect that comorbidity will more and more contribute to shape the health status profile and to affect the prognosis of COPD patients. As a consequence, instruments rating disease-specific health status might lose part of their prognostic value because they have been developed in adult COPD populations free from or with minor comorbidity [3,7]. On the other hand, simple indicators of performance in physical and cognitive domains may predict survival of COPD patients because they are effective in broad elderly populations with multiple chronic diseases [8,9]. For instance, both cognitive impairment and poor physical performance have been associated with worse survival in patients with chronic heart failure [10,11]. Furthermore, being applicable in different populations, performance indexes can provide information on prognosis independently of the main diagnosis, allowing to compare prognosis of patients with different underlying disease.

To date, only a few study are available on the prognostic significance of disease-specific health status indexes such as the St. George Respiratory Questionnaire [12-15], and, to our knowledge, no study has insofar investigated the prognostic value of a multidimensional assessment (MDA), i. e. of an objective rating of different dimensions of health status, in the COPD population. Therefore, we compared a generic MDA and a disease-specific health status measure to evaluate their capacity to provide prognostic information in elderly patients with COPD.

## Methods

### Data source

We used data coming from the Salute Respiratoria nell'Anziano (Respiratory Health in the Elderly, SaRA) study. Between January 1996 and July 1999 a total of 1970 outpatients were recruited from 24 departments of geriatrics or respiratory medicine within the context of the SaRA (Salute Respiratoria nell'Anziano – Respiratory Health in the Elderly) study. Details on the SaRA project are available elsewhere [16]. This is a multi-centre Italian project, investigating various aspects of chronic airway diseases in the elderly population (age ≥ 65 years) attending pulmonary or geriatric outpatient clinics for respiratory and non-respiratory problems. Data from individual centres were collected by a coordinating centre at the Cattedra di Malattie dell'Apparato Respiratorio of the University of Palermo, which was also responsible for the quality control, the retrieval and the final processing of data.

All the patients underwent health status assessment by the disease-specific Saint George Respiratory Questionnaire (SGRQ) [17] and a geriatric MDA covering several areas: social and environmental status, personal history of smoking habit, physical functioning as expressed by the distance walked in 6-minute (WD) [18], cognitive function rated by the Mini Mental Status Examination (MMSE) [19], and mood status assessed by the 15-item Geriatric Depression Scale (GDS) [20].

All the centres were provided with an identical fully computerized water-sealed Stead-Wells spirometer (Baires System; Biomedin; Padua, Italy) that met the standards of the American Thoracic Society (ATS) recommendations for diagnostic spirometry [21]. Pulmonary function tests included baseline spirometry and post-bronchodilator reversibility test performed using fenoterol 100 μg, administered throughout a space chamber. Tests were performed with a standardized technique in all centres and a quality control process was successfully implemented [16].

Patients gave their written consent to participate in the study. The study design was approved by the Ethical Committee of the Coordinating Centre at the University of Palermo, Italy, and by the Ethical Committee of each participating centre.

### Sample selection

From the initial sample, we excluded patients with incomplete spirometric data (N = 212), and also those who had missing data for MDA (N = 122) or SGRQ (N = 10). We further excluded people without bronchial obstruction (FEV1/FVC ≥ 70%, N = 958) and those whose bronchial obstruction might be due to asthma (N = 137) on the basis of a diagnostic algorithm [22]. Thus, the final sample consisted of 531 patients with bronchial obstruction attributable to COPD.

### Follow-up

Vital status at January 30, 2002 was assessed by contacting the registry office of the last municipality of residence, this information was obtained for a total of 449 subjects (84.5%). Causes of death were obtained from the death certificates.

### Analytic approach

We grouped the participants in three groups using both SGRQ and MDA. For disease specific measures we used tertiles of the SGRQ (cut-off values: 2 and 34). For generic measures we used a MDA based score, defined as a combination of depressed mood (GDS ≥ 5) [20], cognitive impairment (MMSE < 24 [23]), and WD < 25% of predicted (all absent: score 0; only one present: score 1; more than one present: score 2).

The agreement of the different classification methods was calculated using the kappa statistic. We analyzed the mortality associated with increasing health status impairment using age- and gender-adjusted Cox regression models. Preliminary analyses showed that the proportional hazard assumption did not hold for SGRQ classes, therefore we decided to model age at death instead of follow-up time. This manipulation made the proportional hazard assumption tenable and also made it unnecessary to include age as a covariate. To evaluate whether any of the two methods could provide information on mortality risk regardless of the severity of obstruction we also repeated the analysis including the forced expiratory volume in the first second, expressed in percent of predicted (FEV1%), as a covariate. Finally, we evaluated the individual contribution to the mortality risk of SGRQ and MDA by analysing them in the same regression model.

All analyses were performed using SAS V9.0 for Windows (SAS Inc., Cary NC).

## Results

We analyzed data from 449 participants (77.3% men, mean age 73.2 years – SD 5.4). Table 1 shows the distribution of demographic characteristics along with pulmonary function parameters, SGRQ, and individual components of the MDA across classes identified by SGRQ and MDA. Average age was similar (about 73 years) in groups obtained by all classification systems. The proportion of men was constant across SGRQ classes, while it linearly decreased as impairment in the MDA increased. Pulmonary function, expressed by the FEV1, worsened with increasing SGRQ or MDA scores. Cognitive function was not associated with the SGRQ, while physical performance and mood status were impaired only in the highest tertile of SGRQ. The agreement of the two classification systems was poor (weighted κ: 0.194), with the best concordance (63%) observed in the group people having MDA score = 0 and being in the first tertile of the SGRQ.

**Table 1:** Demographic and clinical characteristics of participants according to disease severity or health status measures.

	**SGRQ tertile 1**	**SGRQ tertile 2**	**SGRQ tertile 3**
	**(N = 149)**	**(N = 147)**	**(N = 153)**
Mean age (SD)	73.7 (5.9)	73.1 (4.9)	72.9 (5.4)
Males (%)	75.2	76.9	79.7
Mean FEV1 % (SD)	88.7 (19.7)	75.0 (21.1)	52.0 (20.3)
Mean SGRQ score (SD)	0.1 (0.4)	18.3 (9.4)	54.4 (14.0)
Mean WT % (SD)	73.5 (25.1)	76.8 (23.3)	60.2 (26.0)
Mean MMSE score (SD)	27.7 (2.5)	27.5 (2.6)	25.9 (4.2)
Mean GDS score (SD)	3.0 (3.2)	2.8 (2.7)	4.9 (3.3)

	**MDA score 0**	**MDA score 1**	**MDA score 2**
	**(N = 224)**	**(N = 155)**	**(N = 70)**

Mean age (SD)	73.0 (5.2)	73.4 (5.3)	73.6 (6.4)
Males (%)	82.6	74.2	67.1
Mean FEV1 % (SD)	77.4 (25.0)	69.2 (25.2)	59.3 (22.1)
Mean SGRQ score (SD)	16.1 (19.0)	29.8 (24.0)	40.2 (31.1)
Mean WD % (SD)	83.4 (18.8)	64.3 (24.1)	40.1 (17.8)
Mean MMSE score (SD)	28.2 (1.7)	27.0 (2.9)	23.7 (5.2)
Mean GDS score (SD)	1.6 (1.4)	4.9 (3.1)	7.7 (2.7)

**Figure 1:**
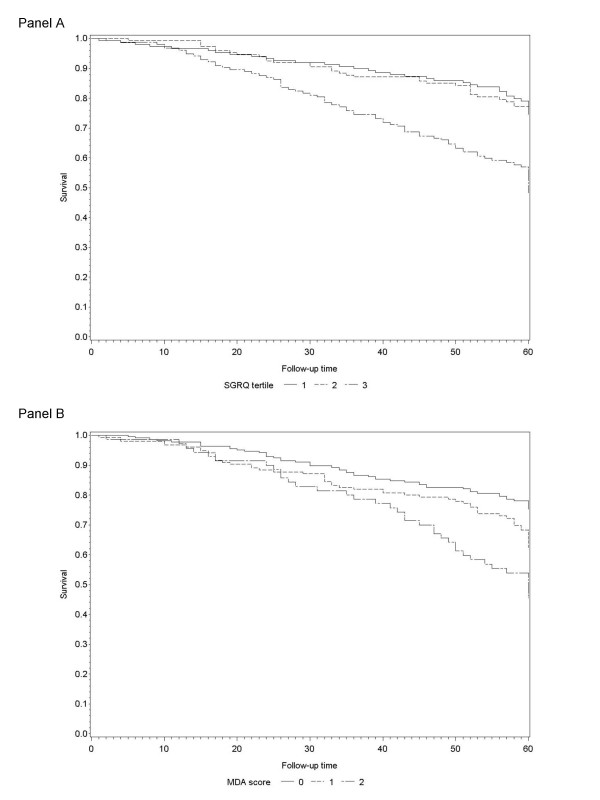
Mortality stratified by SGRQ (panel A) and MDA score (panel B).

Overall, we observed 144 deaths over an average follow-up time of 55 months (median: 61 months, SD: 17.0), with an estimated mortality rate of 6.98/100 person/year (33.9%). The cumulative probability of dying were 29.6%, 22.8%, and 71.8% in people in the first, second and third tertile of the SGRQ, respectively, and 33.5%, 40.4%, and 63.8% for MDA scores 0, 1 and 2, respectively. The relationship between worsening health status, rated by SGRQ or MDA, and mortality after 18 months of follow up is clearly evident in Figure 1.

Twenty out of the 144 deaths were from pulmonary causes (13.4%) and the probability of dying for such causes was 31.3% among those in the upper tertile of the SGRQ score, but only 12.7% among those having a MDA score of 2.

Compared to people in the first tertile of SGRQ score, those in the second tertile had a sex-adjusted HR of 1.22 (0.75 – 1.98) and those in the third tertile of 2.90 (1.92 – 4.40). Adjustment for FEV1, however, caused a decrease in the estimated association, although having a high SGRQ score was still associated with a 86% increase in mortality (95% CI: 1.14 – 3.02). Adjusting for the MDA score, instead, did not cause any important change in the hazard ratio estimates (table 2).

**Table 2:** Hazard ratios for mortality associated with health status measured by SGRQ (upper panel) and MDA (lower panel).

	HR adjusted for age and gender	HR adjusted for age, gender and FEV1%	HR adjusted for MDA score
SGRQ tertile 1	1	1	1
SGRQ tertile 2	1.22 (0.75 – 1.98)	1.04 (0.63 – 1.70)	1.22 (0.75 – 1.98)
SGRQ tertile 3	2.90 (1.92 – 4.40)	1.86 (1.14 – 3.02)	2.68 (1.74 – 4.12)

	HR adjusted for age and gender	HR adjusted for age, gender and FEV1%	HR adjusted for SGRQ class
	
MDA score 0	1	1	1
MDA score 1	1.49 (1.02 – 2.18)	1.25 (0.84 – 1.84)	1.14 (0.77 – 1.69)
MDA score 2	2.01 (1.31 – 3.08)	1.43 (0.91 – 2.23)	1.47 (0.94 – 2.29)

The MDA score also was associated with mortality with a HR of 1.49 (95% CI: 1.02 – 2.18) for people with score = 1 and 2.01 (95% CI: 1.31 – 3.08) for people with score = 2. Among the individual components of the MDA we found the strongest association for the WD (HR: 1.84; 95% CI: 1.31 – 2.59), while depression seems to have lesser importance (HR: 1.55; 95% CI: 1.10 – 2.18) and cognitive impairment was not associated with overall mortality (HR: 0.80; 95% CI: 0.49 – 1.29). After adjustment for severity of obstruction, however, the association between the MDA score and mortality was no more evident (table 2), and among the individual components of the score, only the WD was still associated with the outcome (table 3).

**Table 3:** Mortality rate ratios associated with individual components of the MDA.

	**Adjusted for age and gender**	**Adjusted for age, gender and FEV1%**
WD < 25% predicted	1.84 (1.31 – 2.59)	1.58 (1.12 – 2.22)
MMSE < 24	0.80 (0.49 – 1.29)	0.64 (0.40 – 1.05)
GDS ≥ 5	1.55 (1.10 – 2.18)	1.15 (0.80 – 1.64)

## Discussion

This study shows that a disease-specific health status index and MDA provide different profiles of health status in an elderly COPD population, and only the disease-specific index has prognostic implications when severity of obstruction is taken into account. Since mortality in COPD is related to both respiratory and non respiratory predictors [24,25], the lack of association between mortality and the generic health status index seems to be counterintuitive. Indeed, most (120/144) of the deaths in our sample were attributed to non-respiratory causes, and conditions such as chronic renal failure or cardiovascular diseases carry important prognostic implications in COPD populations and have a well known disabling potential which MDA is more likely to capture than SGRQ [26]. However, while all the components of our MDA score were expected to individually predict mortality, only for the 6' WT this association was present after adjustment for the severity of obstruction, confirming the prognostic value of 6'WT [25]. Depression and cognitive impairment, instead, could in part capture the increased risk for mortality linked to the severity of obstruction, but they do not carry an excess risk of mortality by themselves.

Depression has an obvious impact on quality of life in COPD patients [27] but, while it is associated with excess mortality in the general elderly population [28], only in two studies it has been found to be negatively associated with survival [12,29] in people with COPD. These data, however, come from people with severe or exacerbated COPD, and this can explain the different finding of our study, in which the association between depression and mortality is not independent of bronchial obstruction.

Overall cognitive impairment is an important risk factor for mortality in the general elderly population [30], but in the COPD population it has been shown that only the impairment in specific cognitive tasks, namely executive and praxic, are associated with mortality, but not impairment in the overall cognitive performance or in non-executive tasks [9,31]. Another factor that may explain the lack of association between cognitive impairment and mortality is that very severe COPD, which is frequently complicated by clinically meaningful cognitive dysfunction [32], was underrepresented in our population. Furthermore, we studied only patients with a high quality spirometry, automatically excluding thus most of cognitively deteriorated patients [16,33].

We confirm the association of the SGRQ score with mortality reported by other investigators [12-15], but in a sample more representative of the general elderly COPD population. In fact, current knowledge on the prognostic value of the SGRQ was obtained either in samples excluding women [13,15] or in samples of COPD patients recruited in the hospital setting [12,14]. The association we found, however, was evident only for people in the upper tertile of this score, indicating a poor discriminative capacity of this index. Indeed, a bending point in the decline of the SGRQ is evident only for FEV1 < 50% [34].

Women prevailed in the worst MDA category, while their prevalence did not distinguish SGRQ tertiles. This finding suggests that MDA captures health status dimensions which are more sensible to deterioration in women. Indeed, it has been demonstrated that in a broad elderly population disability and depression are more prevalent and incident among women [35,36]. Furthermore, prevalence of depressive symptoms has been reported to be higher among women than among men with COPD [37]. Finally, non-respiratory factors seem to be related to mortality in women, but non in men, with COPD [38]. Accordingly, MDA classificatory and discriminative properties are expected to improve for increasing prevalence of females in the COPD populations. Furthermore, older age is associated with non respiratory comorbility, and this might further enhance the classificatory role of MDA [39,40].

This study has several limitations. First, GOLD 3–4 stages were underrepresented in our population. Accordingly, a somewhat different set of MDA components might be more suited for a more diseased COPD population, while present conclusions should be considered to apply to the average ambulatory COPD population. Second, likely due to the confinement of smoke-related disease to young and adult women up to now, the female/male ratio (1:3.5) in our study was lower than that reported (1:2) in the general population [41]. Third, about 16% of participants were lost to follow-up and therefore our results may be biased if reasons for being lost were associated with group membership. Finally, causes of deaths came from death certificates, and therefore some misclassification is possible with regards to pulmonary deaths.

## Conclusion

This study shows that in an elderly COPD population a disease-specific health status measure, the SGRQ, can predict mortality independently of severity of bronchial obstruction, although without a clearly linear relationship. On the contrary, the MDA could predict mortality only when the severity of obstruction was not taken into account, indicating that its dimensions are affected by the severity of diseases but are not independently linked to mortality. Our data support the conclusion that disease-specific health status measures may be more useful as a prognostic tool than generic instruments in an elderly population with mild-moderate COPD.

## Competing interests

The authors declare that they have no competing interests.

## Authors' contributions

MEC participated in data analysis and interpretation, drafted the manuscript and reviewed it for important intellectual contents. CP planned the statistical analysis and performed it, participated in manuscript drafting and reviewed the paper for important intellectual contents. FF collected the follow-up information and revised the manuscript for important intellectual contents. VB participated in study design and data collection, and revised the manuscript for important intellectual contents. RA–I had the original idea for the study, participated in its design and in drafting the manuscript, and revised the paper for important intellectual content. All authors read the final version of the manuscript and approved it for publication.

## Pre-publication history

The pre-publication history for this paper can be accessed here:


